# Nitro‐oxidation of BK_Ca_ mediates neuro‐ and micro‐vascular dysfunction in female *5x‐FAD* mice

**DOI:** 10.1002/alz.090854

**Published:** 2025-01-03

**Authors:** Josiane Fernandes da Silva, Felipe Davis Polk, Stephenie H. Thai, Ella C. Frank, Paige E. Martin, Paulo Pires

**Affiliations:** ^1^ University of Arizona, Tucson, AZ USA

## Abstract

**Background:**

Cerebral microvascular dysfunction and nitro‐oxidative stress are present in patients with Alzheimer’s disease (AD) and may contribute to disease progression and severity. A pro‐nitro‐oxidative environment can lead to post‐translational modifications of ion channels central to microvascular regulation in the brain, including the large conductance Ca^2+^‐activated K^+^ channels (BK_Ca_). Nitro‐oxidative modulation of BK_Ca_ can resulting in decreased activity and vascular hyper‐contractility, thus compromising neurovascular regulation. We hypothesized that nitro‐oxidation of vascular BK_Ca_ function blunts micro‐ and neuro‐vascular responses in *5x‐FAD* mice.

**Methods:**

Six‐months‐old female *5x‐FAD* and wild‐type (WT) littermates were used. Brain lysates were used to quantify BK_Ca_ nitro‐oxidation. Cerebral arteries lysates were used to assess BK subunit expression by qPCR. A different set of cerebral arteries were used for patch clamp electrophysiology assessment of BK_Ca_ currents, *ex vivo* pressure myography and quantification of calcium (Ca^2+^) sparks in smooth muscle cells (SMC) by spinning‐disk confocal microscopy. Basal cerebral perfusion and neurovascular coupling were assessed by laser speckle contrast imaging. Differences between means were tested using t‐tests and considered statistically significant when P<0.05.

**Results:**

We observed that BK_Ca_ S‐nitrosylation, a proxy for nitro‐oxidative modification, was increased in the brain of AD patients and in *5x‐FAD* females. In functional experiments, we observed that BK_Ca_ currents were lower in cerebral artery SMC of *5x‐FAD* than in WT. As a consequence, microvascular reactivity to BK_Ca_ blockade (iberiotoxin, 30 nM) was blunted in *5x‐FAD*, suggesting lower basal BK_Ca_ activity. This reduction in microvascular BK_Ca_ activity was independent of SMC Ca^2+^ sparks or BK_Ca_ mRNA expression. We then performed rescue experiments by incubating membrane patches and pressurized arteries with the reducing agent dithiothreitol (DTT, 10 µM), and observed that DTT restored BK_Ca_ currents and sensitivity to iberiotoxin in cerebral artery SMC from *5x‐FAD*. *In vivo* experiments showed that basal perfusion to the frontal cortex and neurovascular coupling were lower in *5x‐FAD* when compared to WT littermates.

**Conclusion:**

These data suggest that excessive nitro‐oxidation of BK_Ca_ mediates neuro‐ and micro‐vascular impairments in female *5x‐FAD* mice, and that this process is reversible and can be rescued by reducing agents.

**Funding**: National Institutes on Aging and Alzheimer’s Association

